# Circulating Proteomic Profiles in Ambulatory Patients With Advanced Stage C2D Heart Failure: Insights From the Registry Evaluation of Vital Information for VADs in Ambulatory Life (REVIVAL) Registry

**DOI:** 10.1016/j.cardfail.2026.01.006

**Published:** 2026-01-22

**Authors:** DOUGLAS L. MANN, JULIO A. CHIRINOS, JINSHENG YU, WEI YANG, DENNIS M. MCNAMARA, BLAIR RICHARDS, WENDY C. TADDEI-PETERS, NEAL JEFFRIES, GARRICK C. STEWART, CHENAO QIAN, THOMAS P. CAPPOLA, KEITH D. AARONSON

**Affiliations:** 1 Washington University School of Medicine, St. Louis, Missouri; 2 Division of Cardiovascular Medicine, Hospital of the University of Pennsylvania, Philadelphia, Pennsylvania; 3 University of Pennsylvania Perelman School of Medicine, Philadelphia, Pennsylvania; 4 University of Pittsburgh Medical Center, Pittsburgh, Pennsylvania; 5 University of Michigan, Ann Arbor, Michigan; 6 National Heart Lung and Blood Institute, Bethesda, Maryland; 7 Brigham and Women’s Hospital, Boston, Massachusetts.

## Introduction

Recent studies underscore the need to identify patients with ambulatory stage C heart failure (HF) who, despite receiving guideline-directed therapy (GDMT), experience persistent symptoms and recurrent hospitalization, which is associated with high risk for near-term progression to stage D HF (“C2D” HF) and the need for a left ventricular assist device (VAD) or cardiac transplantation.^1^ The ability of traditional clinical risk models and biomarkers to predict freedom from HF events in patients with C2D HF remains incompletely understood and continues to evolve.^2^ The Registry Evaluation of Vital Information for VADs in Ambulatory Life (REVIVAL) was a prospective, multicenter registry of high-risk, ambulatory patients with advanced HF. Despite high use of GDMT and specialized HF care, 2-year follow-up revealed significant rates of HF events, death, urgent transplantation, or durable left VAD implantation, with mortality of 8% at 1 year and 16% at 2 years.^3^

In contrast to single HF biomarkers, which primarily reflect myocardial stretch or injury and fail to capture the biological complexity of advanced HF,^2^ modern proteomic platforms enable simultaneous measurement of hundreds of proteins, offering a broader view of disease mechanisms and improved risk stratification. To explore the biological mechanisms associated with progression from stage C to stage D heart failure, we performed proteomic profiling in a subset of participants from the REVIVAL registry, with independent validation in the Penn Heart Failure Study (PHFS).^4^

## Methods and results

### Study Population

We analyzed a subset of participants from the REVIVAL registry. Two cohorts with INTERMACS 5–7 profiles were identified. Group A (n = 39) included patients event-free at 6 months, whereas Group B (n = 39) comprised patients with death, urgent transplant, VAD, or HF hospitalization by 6 months. Groups were matched by age, sex, and race and were well balanced overall, except for a greater prevalence of diabetes in group B (51% vs 26%) (Table 1). Patients were well treated with GDMT and device therapy for HF with reduced ejection fraction. The validation cohort included patients from the PHFS. To mirror REVIVAL, 843 patients with New York Heart Association (NYHA) class III–IV from PHFS were identified, yielding 318 cases and 318 controls selected using 1:1 propensity-score nearest neighbor matching without replacement on the basis of NYHA class (III vs IV), sex, age (±5 years), race, and baseline inotropic support.

### Plasma Proteomic Profiling

Baseline REVIVAL serum samples were analyzed using the SomaScan (version 1.0; SomaLogic, Boulder, CO), which identifies 1305 unique protein targets, as described.^5^ For the PHFS, we used the SomaScan assay, version 4, which includes 4979 modified aptamer reagents for 4776 unique protein targets, including all aptamers primarily assessed in REVIVAL.

### N-Terminal Pro B-Type Natriuretic Peptide (NT-proBNP)

Baseline measurements of NT-proBNP were assayed using a Meso Scale Discovery (MSD, Rockville, MD) singleplex R-PLEX electrochemiluminescent sandwich immunoassay.

### Clinical Outcomes

**Fig. 1.**
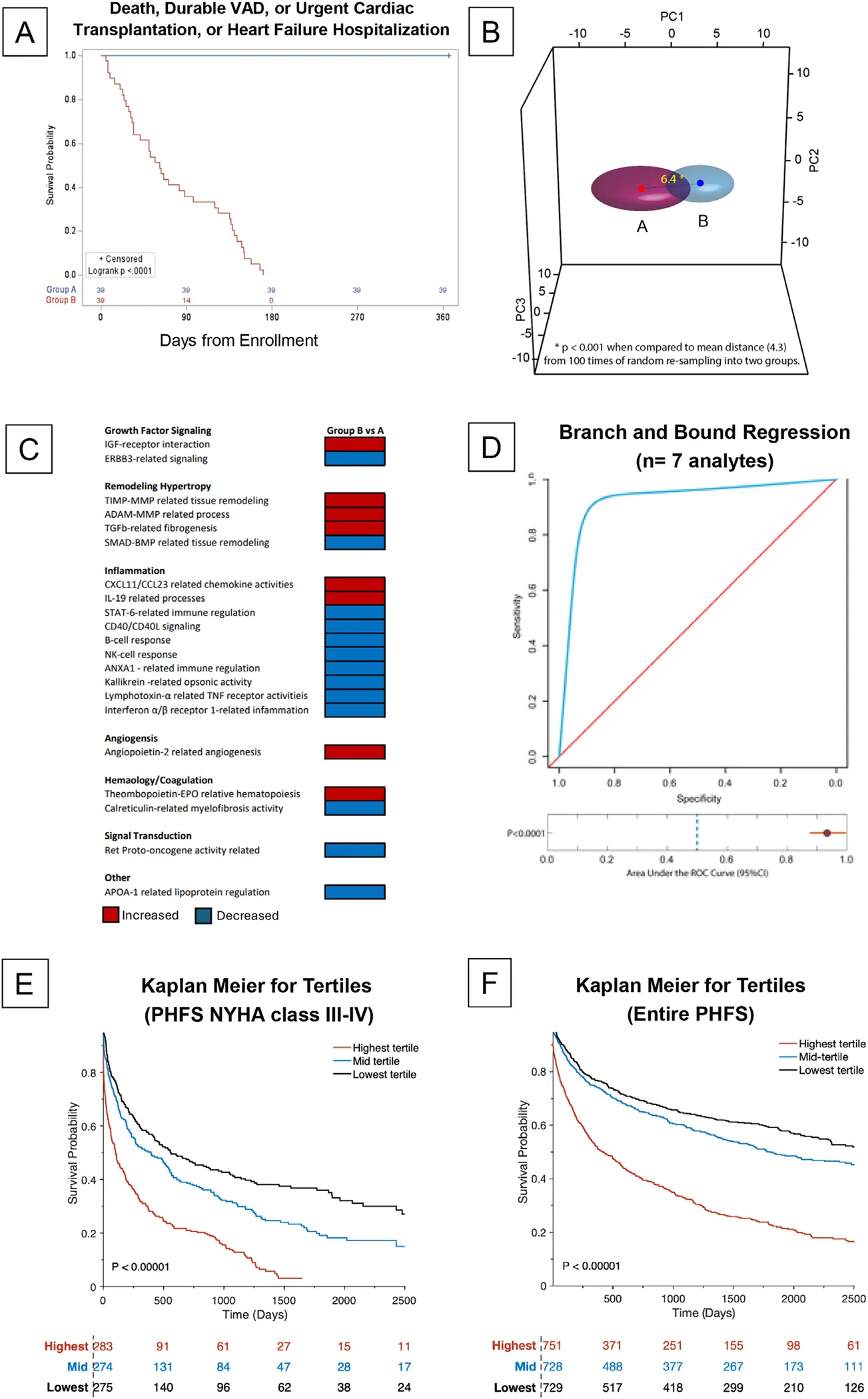
(A) Kaplan-Meier analysis of death, urgent transplantation, durable VAD implantation, and HF hospitalization in the REVIVAL proteomic substudy through 365 days. (B) Principal component analysis–based spatial relationship between patients in groups A and B. (C) Enriched biological themes were generated by COMPBIO from differentially expressed proteins between group A and group B, which were grouped into higher-level biological groupings in order to permit comparisons between the groups. (D) Receiver operator curves for the model performance of classifiers identified from the classification analysis using a Branch-and-Bound regression analysis on the basis of 7 analytes (see Results). (E) Kaplan-Meier curves for incident death, transplantation, VAD implantation, and HF hospitalization in PHFS NYHA class III-IV participants using the 7 analytes identified in the REVIVAL cohort. There was a marked difference in the risk of the endpoint between the 3-risk score tertiles (*P* < .0001). (F) Kaplan-Meier curves for incident death, transplantation, VAD implantation, and HF hospitalization in the entire PHFS cohort with available proteomics data using the 7 analytes identified in the REVIVAL cohort, stratified by tertiles of the risk score. There was a marked difference in the risk of the endpoint between the 3-risk score tertiles (*P* < .0001), especially among participants in the highest risk score tertile. HF, heart failure; NYHS, New York Heart Association; PHFS, Penn Heart Failure Study; REVIVAL, Registry Evaluation of Vital Information for VADs in Ambulatory Life; VAD, ventricular assist device.

Figure 1A shows the Kaplan-Meier curves for the composite of death, urgent transplantation, left VAD implantation, and HF hospitalization. Through 365 days of follow-up, there were no HF events in patients in group A, whereas 100% of patients in group B had a HF hospitalization, urgent transplantation (n = 5), durable VAD implantation (n = 13), and HF hospitalization (n = 33).

### Proteomic Profiling of Patients With Advanced HF

Of 1305 protein analytes measured, 132 were differentially expressed between groups A and B (false-discovery rate *P* < .05). Among the differentially expressed proteins, 59 were increased, and 73 decreased in group A when compared with group B. Principal component analysis of these proteins demonstrated 2 partially overlapping clusters (Fig. 1B). A pseudo–heat map of the differentially expressed proteins was generated using COMPBIO.^5^ Proteins increased in group B were enriched in pathways related to growth factor signaling, cardiac remodeling, and angiogenesis. In contrast, patients with adverse clinical outcomes exhibited decreased expression of themes linked to innate and adaptive immune responses, as well as lipid metabolism, highlighting distinct proteomic signatures associated with disease trajectory (Fig. 1C).

### Analysis of Classification Models for Predicting Clinical Outcomes

To predict death, urgent transplantation, or durable left VAD implantation, penalized logistic regression models were applied to the 132 differentially expressed proteins. Receiver operating characteristic curves assessed the predictive accuracy of single-protein and multiprotein models. Three regularized approaches were used: LASSO (Least Absolute Shrinkage and Selection Operator), elastic net, and ridge regression. LASSO identified a single analyte, basal cell adhesion molecule (BCAM), with an area under the curve (AUC) of 0.74. Elastic net identified 26 analytes (AUC 0.84), whereas ridge regression using all 132 analytes showed poor discrimination (AUC 0.57). To derive a parsimonious, clinically applicable panel, branch-and-bound optimized logistic regression was applied,^6^ identifying panels of 6–11 proteins with AUCs of 0.88–0.92. The optimal model comprised 7 analytes, including BCAM (CD239), CD36, SPINT1, PLG, PPY, CXCL13, and HGF, with an AUC of 0.92 (Fig. 1D). In contrast, the AUC for NT-proBNP was 0.75, which was comparable with the AUC from the LASSO regression model with a single analyte (BCAM).

### PHFS Validation Cohort

In the PHFS validation cohort, the composite REVIVAL proteomic risk score predicted case/control status with an AUC of 0.60 (95% confidence interval [CI], 0.55–0.64; *P* < .0001) and a Harrell C-statistic of 0.59 (95% CI, 0.56–0.62), indicating reduced performance of the model. This likely reflects differences in HF populations and risk stratification tools: REVIVAL was enriched for high-risk patients, Interagency Registry for Mechanically Assisted Circulatory Support (INTERMACS) 5–7, who experienced HF events within 6 months, whereas the PHFS included patients with NYHA class III–IV HF; importantly, INTERMACS profiling provides superior prognostic discrimination compared with the NYHA class-–based classification.^7^ As shown in Figure 1E, Cox regression confirmed the REVIVAL proteomic risk score as a significant predictor of the composite endpoint of death, HF hospitalization, urgent transplant, or durable VAD implantation (hazard ratio per standard deviation increase=1.23; 95% CI, 1.16–1.31; *P* < .0001). Kaplan-Meier analyses in NYHA III–IV participants demonstrated clear risk separation by tertiles (*P* < .0001). To assess generalizability, the model was tested in the entire PHFS cohort (NYHA I–IV; n=2248), where it predicted outcomes with hazard ratio per standard deviation increase=1.15 (95% CI, 1.12–1.19; *P* < .0001) and a Harrell C-statistic of 0.61 (95% CI, 0.59–0.63). Tertile-based stratification again showed marked differences (*P* < .0001), with the greatest-risk individuals experiencing substantially worse outcomes (Fig. 1F).

## Discussion

The major finding of this study is that circulating protein profiles in clinically stable patients with advanced HF and INTERMACS 5–7 profiles differ significantly from those of patients with similar INTERMACS profiles who develop clinical instability or require left VAD implantation or cardiac transplantation within 6 months. Bioinformatic analysis identified a composite REVIVAL proteomic risk panel comprising 7 proteins, the majority of which have not been linked to the pathogenesis of HF, save for CXCL13 and HGF. Remarkably, 5 proteins were linked to extracellular matrix (ECM) remodeling and fibrosis (Table 2), suggesting that biomarker panels associated with ECM dynamics may identify high-risk, vulnerable patients with C2D HF. Unlike NT-proBNP and Troponin T, which reflect hemodynamic stress and myocardial injury, respectively, biomarkers that are associated with progressive fibrotic ECM remodeling may persist, despite normalization of hemodynamic load or resolution of cardiac injury. It is also possible that ECM remodeling biomarkers could be paired with advanced cardiac imaging capable of detecting subclinical adverse left ventricular chamber remodeling to improve risk stratification in vulnerable populations. Although these results offer potential mechanistic insights into progression from stage C to stage D heart failure, they should be regarded as hypothesis-generating and validated in larger, prospective C2D HF cohorts.

## Figures and Tables

**Table 1:** Clinical characteristics at baseline

	Group A (No Events; n = 39)	Group B (Early Events; n = 39)	Total (n = 78)	*P*

Age, y	62 (56–68)	61 (54–69)	62 (56–68)	.84
Female	9 (23%)	9 (23%)	18 (23%)	1
African American	7 (18%)	7 (18%)	14 (18%)	1
Hispanic/Latino	4 (10%)	3 (8%)	7 (9%)	1.0*
Ischemic etiology	16 (41%)	13 (33%)	29 (37%)	.48
Diabetes mellitus	10 (26%)	20 (51%)	30 (38%)	.02
Atrial fibrillation	5 (13%)	7 (18%)	12 (15%)	.53
BMI, kg/m^2^	29.3 (24.5–33)	29.4 (25.5–35.6)	29.4 (25.5–34)	.73
Systolic blood pressure, mm Hg	102 (96–116)	106 (98–118)	105 (98–116)	.68
Heart rate, bpm	74 (68–82)	73 (69–80)	74 (69–81)	.92
Creatinine, mg/dL	1.3 (1.1–1.5)	1.4 (1.2–1.8)	1.3 (1.1–1.6)	.11
Ejection fraction, %	20 (20–25)	20 (17–25)	20 (18–25)	.24
Heart failure severity				
INTERMACS profile				.66*
4	2 (5%)	2 (5%)	4 (5%)	
5	9 (23%)	8 (21%)	17 (22%)	
6	10 (26%)	15 (38%)	25 (32%)	
7	18 (46%)	14 (36%)	32 (41%)	
Medical and electrical therapies				
Beta-blocker	38 (97%)	35 (90%)	73 (94%)	.36*
ACE inhibitor/ARB/ARNI	35 (90%)	30 (77%)	65 (83%)	.13
Hydralazine plus nitrate	6 (15%)	6 (15%)	12 (15%)	1.00
Mineralocorticoid receptor antagonist	32 (82%)	29 (74%)	61 (78%)	.41
CRT-D or ICD	39 (100%)	39 (100%)	78 (100%)	–

Values reported as median (25th–75th percentile), orn (%); *P* values are *χ*^2^ or * Fisher exact test or Wilcoxon rank sum *P* values.

ACE, angiotensin-converting enzyme; ARB, angiotensin receptor blocker; ARNI, Angiotensin receptor neprilysin inhibitor; BMI, body mass index; CRT, cardiac resynchronization therapy; GFR, glomerular filtration rate; ICD, implantable cardioverter defibrillator; INTERMACS, Interagency Registry for Mechanically Assisted Circulatory Support; NYHA, New York Heart Association.

**Table 2:** Biological function of proteomic risk factors and their role in heart failure (HF)

Protein	Cellular Function	Reported in HF

Basal cell adhesion molecule (BCAM)	A glycoprotein involved in cell adhesion and interaction with the extracellular matrix. BCAM has been linked with several fibrotic diseases, including progression of idiopathic pulmonary fibrosis (IPF)	No
Cluster of differentiation 36 (CD36)	CD36 is expressed on the surface of cardiomyocytes and endothelial cells. CD36 binds to thrombospon-din-1 (TSP-1), a key activator of latent transforming growth factor (TGF)-*β*, which promotes profibrotic signaling pathways.	Yes. Increased in diabetic cardiomyopathy and atherosclerosis. Decreased expression in ischemia-reperfusion injury.
Serine peptidase inhibitor, Kunitztype 1 (SPINT1)	Regulates cell signaling related to tissue repair and inflammation and epithelial-to-mesenchymal transition (EMT), which is a key process in fibrosis. Also inhibits proteases that contribute to extracellular matrix (ECM) degradation.	No
Plasminogen (PLG)	Involved in fibrinolysis and clot resolution. However, also has noncanonical functions that are critical in cardiac repair mechanisms, including EMT, ERK phosphorylation, and TGF-*β* activation. Also influences inflammation and ECM remodeling through its activation of matrix metalloproteinases (MMPs) and chemokine pathways.	Yes. Genetic disruption of PLG abolishes wound healing in mice after myocardial infarction
Pancreatic peptide (PPY)	Produced and secreted by pancreatic peptide cells in the pancreas. Plays a regulatory role in digestion and metabolism. PPY may play a role in energy homeostasis and has been shown to influence energy expenditure and fat storage.	No
C-X-C motif chemokine ligand (CXCL13)	A chemokine involved in immune cell recruitment, particularly B cells. CXCL13 has also been linked to T-cell activation, monocyte activation, and B-cell trafficking. Contributes to the fibrotic niche by promoting fibroblast activation and ECM deposition.	Yes. CXCL13 participates in ECM regulation. Mice with genetic deletion of CXCR5 (receptor for CXCL13) had increased mortality, LV dilatation, and enhanced matrix remodeling during cardiac pressure overload. Patients with HF have increased myocardial levels of CXCR5 reversed after left ventricular assist device (LVAD) support.
Hepatocyte growth factor (HGF)	Plays a role in cell growth, tissue repair, and angiogenesis in various cell types.	Yes. Elevated serum levels of HGF are associated with greater rates of mortality in patients with acute and advanced heart failure. HGF levels provide independent and additive prognostic information to N-terminal pro B-type natriuretic peptide (NT-proBNP).
